# Prospective assessment of a score for assessing basic critical-care transthoracic echocardiography skills in ventilated critically ill patients

**DOI:** 10.1186/2110-5820-4-12

**Published:** 2014-04-27

**Authors:** Mathieu Jozwiak, Xavier Monnet, Raphaël Cinotti, Fréderic Bontemps, Jean Reignier, Guillaume Belliard

**Affiliations:** 1 Centre Hospitalier Départemental de la Vendée, service de réanimation, La Roche-sur-Yon F-85000, France; 2 AP-HP, Hôpitaux Universitaires Paris-Sud, Hôpital de Bicêtre, service de réanimation médicale, Le Kremlin-Bicêtre F-94270, France; 3 Faculté de Médecine Paris-Sud, Université Paris-Sud, EA4533, Le Kremlin-Bicêtre F-94270, France; 4 Centre Hospitalo-Universitaire Guillaume et René Laennec, service de réanimation chirurgicale, Nantes F-44000, France; 5 Centre Hospitalier Côte de Lumière, service de médecine polyvalente, Les Sables d’Olonne F-85100, France; 6 Centre Hospitalier Bretagne Sud Lorient, service de réanimation médicale, Lorient F-56100, France

**Keywords:** Goal-oriented bedside echocardiography, Basic critical-care echocardiography skills, Non-cardiologist intensivists, Hemodynamic assessment, Intensive care unit

## Abstract

**Background:**

We studied a score for assessing basic transthoracic echocardiography (TTE) skills exhibited by residents who examined critically ill patients receiving mechanical ventilation.

**Methods:**

We conducted a prospective study in the 16 residents who worked in our medical-surgical ICU between 1 May 2008 and 1 November 2009. The residents received theoretical teaching (two hours) then performed supervised TTEs during their six-month rotation. Their basic TTE skills in mechanically ventilated patients were evaluated after one (M1), three (M3), and six (M6) months by two experts, who used a scoring system devised for the study. After scoring, residents gave their hemodynamic diagnosis and suggested a treatment.

**Results:**

The 4 residents with previous TTE skills obtained a significantly higher total score than did the 12 novices at M1 (18 (16 to 19) versus 13 (10 to 15), respectively, *P* = 0.03). In the novices, the total score increased significantly during training (M1, 13 (10 to 14); M3, 15 (12 to 16); and M6, 17 (15 to 18); *P* < 0.001) and correlated significantly with the number of supervised TTEs (r = 0.68, *P* < 0.0001). In the overall population, agreement with experts regarding the diagnosis and treatment was associated with a significantly higher total score (17 (16 to 18) versus 13 (12 to 16), *P* = 0.002). A total score ≥ 19/20 points had 100% specificity (95% confidence interval, 79 to 100%) for full agreement with the experts regarding the diagnosis and treatment.

**Conclusions:**

Our results validate the scoring system developed for our study of the assessment of basic critical-care TTE skills in residents.

## Background

Echocardiography is increasingly used to evaluate the hemodynamic status of patients in the ICU or operating room. Transthoracic echocardiography (TTE) and transesophageal echocardiography (TEE) provide a broad range of cardiovascular parameters for rapidly understanding the causes and mechanisms of circulatory failure [1-8]. In many studies, echocardiography was found to contribute substantially to the goal-oriented treatment of circulatory failure [9-14]. Both TTE and TEE used as tools for hemodynamic management benefited therapeutic decisions in patients receiving intensive care [15-21] or undergoing surgery [22-25]. Echocardiography is therefore among the recommended tools for managing patients with shock [26].

The main limitation of echocardiography is that an experienced operator is required. Formal teaching programs are a crucial prerequisite to the development of ultrasound techniques in critical care [27,28]. Experts agree that basic critical-care echocardiography skills must be taught to all intensivists [29]. Several studies have established that non-cardiologist intensivists can acquire these basic skills after a short period of training [9-11,30-35].

Determining whether trainees have acquired the necessary echocardiography skills is a major issue. There is no consensus about the number of supervised echocardiograms a trainee must perform to become sufficiently skilled [29]. A standard scoring system with a cutoff value indicating that basic skills have been acquired would be helpful [36]. Such a scoring system has been validated for TEE in critically ill patients [37]. A recent study that used this score in ICU intensivists showed that 31 supervised TEEs in mechanically ventilated ICU patients were needed to reliably achieve competence in advanced critical-care TEE [38]. To our knowledge, these two studies are the only ones to use such a standard scoring system. However, both studies focused on TEE, although TTE is often the first-line ultrasound method used for hemodynamic assessment [36]. Moreover, neither study evaluated whether higher scores predicted better treatment decisions by the trainees. Finally, the scoring system used in these studies includes quantitative measurements, whereas basic critical-care echocardiography involves only semi-quantitative measurements [29].

Here, our objective was to validate a score for assessing basic TTE skills in trainees examining mechanically ventilated ICU patients. We also identified the score cutoff above which trainees made appropriate decisions regarding hemodynamic management.

## Methods

### Study protocol

We conducted a prospective observational study in the 15-bed medical-surgical ICU of a general hospital between 1 May 2008 and 1 November 2009. The protocol for standard management of circulatory failure was the protocol used routinely in our ICU. Standard management involved routine TTE at circulatory failure onset and as needed thereafter. In particular, no other devices are used in our ICU to assess hemodynamic status of the patients. No changes in the standard management of circulatory failure occurred in the ICU over the study period. Therefore, according to French law on medical research, informed consent from the patients or next of kin was not required for this observational study.

### Residents

We included the 16 residents who worked in the ICU during the study period. Their specialties were anesthesiology (n = 6), cardiology (n = 2), emergency medicine (n = 4), and other (n = 4). They had previously completed 5 ± 2 rotations of 6 months each. We divided the residents into two groups: novices (n = 12) had no theoretical and/or practical experience with TTE, whereas skilled residents (n = 4) were cardiologists or certified echocardiographers.

### Training

TTE was performed using a Sequoia Siemens C-512 (Siemens Medical Solutions, Malvern, PA, USA) equipped with an Acuson 3V2c probe (Acuson Corp, Mountain View, CA, USA). Training was supervised by two board-certified echocardiographers with at least two years of experience at level-three competence according to the American Society of Echocardiography [39]. All residents received a two-hour theoretical teaching on TTE, designed to show them a selection of typical TTE recordings, during the first week of their six-month rotation in the ICU. Then, residents followed the hands-on training session by performing TTEs repeatedly during their six-month rotation in the ICU under the supervision of one of the two experts, who provided teaching as appropriate. For this purpose, the residents performed all TTEs ordered by the attending physician in mechanically ventilated (volume assist-controlled mode) patients with acute circulatory failure defined as systolic arterial pressure ≤ 90 mmHg despite adequate fluid resuscitation [26], admitted during their six-month rotation in the ICU. Residents could perform several TTE examinations on the same patient during their hands-on training sessions. As far as possible, patients were placed in left lateral decubitus for TTE examination. Otherwise, they were placed in the 30° semi-recumbent position according to the standard management of the patients in our unit. The use of sedatives and neuromuscular blockers was at the discretion of the attending physician. We chose to investigate only mechanically ventilated patients in order to standardize the conditions under which TTE was performed.

### Evaluation of the residents and scoring system

After one (M1), three (M3), and six (M6) months, the residents were evaluated during a TTE examination involving the acquisition of apical, subcostal, and parasternal views. Patients with poor echogenicity during TTE, as assessed by the experts, were not considered for the evaluation. The TTE used for the evaluation was performed in a patient who was not among the resident’s patients and on whom the residents had never previously performed TTE examination during their hands-on training sessions. Nevertheless, the resident could have access to all patients’ medical records at any time.

At each study time point, the residents were evaluated by both experts using the scoring system detailed in Table 1. The echocardiographic key-findings the trainees should be able to identify consisted of a real time evaluation. Then, the residents gave their hemodynamic diagnosis and suggested a treatment. Based on their TTE examination and on the data in the patient’s record, residents had to choose among the following hemodynamic diagnoses: hypovolemia in the case of large respiratory variation in inferior vena cava diameter; impaired left ventricular ejection fraction, and/or right ventricular failure in the case of marked dilation of the right ventricle. They also chose among the following therapeutic options, according to the therapeutic algorithm used in our ICU: volume expansion in the case of hypovolemia, vasopressors when there was right ventricular failure or when the acute circulatory failure could neither be attributed to hypovolemia nor to left ventricular dysfunction, and/or inotropes in the case of left ventricular failure. TTE was then repeated by one of the two experts, who assessed the quality of the resident’s TTE and the relevance of the proposed diagnosis and treatment.

**Table 1:** Scoring system used to evaluate transthoracic echocardiography skills

	**0**	**2**	**3**	**Total score**
**Quality of TTE views**				
Left parasternal long axis	Not recorded	Not optimal	Optimal	/2
Left parasternal short axis	Not recorded	Not optimal	Optimal	/2
Apical four-chamber view	Not recorded	Not optimal	Optimal	/2
Apical two-chamber view	Not recorded	Not optimal	Optimal	/2
Subcostal four-chamber view	Not recorded	Not optimal	Optimal	/2
IVC view	Not recorded	Not optimal	Optimal	/2
				/12
**Semi-quantitative measurements**^**a**^				
Right ventricular dilation	Disagreement for presence	Agreement for presence but disagreement for degree	Agreement for presence and degree	/2
Pericardial effusion	Disagreement for presence	Agreement for presence but disagreement for degree	Agreement for presence and degree	/2
Respiratory variation in IVC diameter	Disagreement for presence	Agreement for presence but disagreement for degree	Agreement for presence and degree	/2
Left ventricular ejection fraction	Disagreement for presence	Agreement for presence but disagreement for degree	Agreement for presence and degree	/2
				/8
				/20

^a^disagreement or agreement with the assessment of two experts.

*IVC*: inferior vena cava; *TTE*: transthoracic echocardiography.

The scoring system was based upon the one developed previously for TEE [37] but included neither quantitative measurements nor scoring of the diagnosis and treatment. As shown in Table 1, the first part of the score assessed the quality of the TTE views, which was rated as follows: 0, view not obtained; 1, view not considered optimal by the experts; and 2, view considered optimal by the experts. The second part of the score assessed the semi-quantitative measurements needed to answer the clinical questions relevant to basic critical-care echocardiography, as defined when the study was designed [27,40]: right ventricular dilation (none, moderate, marked), pericardial effusion (none, non-compressive, compressive), respiratory variation in inferior vena cava diameter (none, minimal, large), and visual estimation of the left ventricular ejection fraction (normal, decreased, markedly decreased) (Table 1). We considered that right ventricular dilation was marked when its area was greater than or equal to the area of the left ventricle [41]. We considered that respiratory variation in inferior vena cava diameter was large when there was a marked visual inspiratory increase in inferior vena cava diameter assessed in M-mode. Each semi-quantitative measurement was rated as follows: 0, disagreement with the experts regarding presence of the abnormality; 1, agreement with the experts regarding presence but disagreement regarding severity of the abnormality; and 2, full agreement with the experts. The maximum total score was 20 (Table 1). The hemodynamic diagnosis and suggested treatment together was rated 1 when fully consistent with the experts’ conclusions and as 0 otherwise. TTE scoring and rating of the diagnosis and suggested treatment were performed offline by both experts working together. The time from the beginning of TTE and suggestion of a treatment was recorded.

### Statistical analysis

Normality of data distribution was tested using the Kolmogorov-Smirnov normality test. Variables were summarized as percentages, means and standard deviations, or medians (25% to 75% interquartile range (IQR)), as appropriate.

The total score was compared between novices and skilled residents at M1 using the Mann-Whitney *U*-test. Within the novice group, we compared data at M1, M3, and M6 using Friedman’s test for quantitative variables and the chi-square McNemar’s test for qualitative variables. The correlation between scores and number of supervised TTEs was assessed using a logarithmic regression model.

Residents were separated into two groups based on whether their diagnosis/treatment rating was 1 or 0 (agreement versus disagreement with the experts), and the scores were compared between these groups using the Mann-Whitney *U*-test. In the overall population of novices and skilled residents, we plotted a receiver-operating characteristics (ROC) curve to identify the score cutoff that predicted full agreement between residents and experts regarding the diagnosis and treatment (rating of 1). The best cutoff was defined as the value providing the highest Youden index. Sensitivity and specificity were computed as means (95% confidence interval (CI)).

*P* values < 0.05 were considered significant. Statistical tests were performed using Medcalc 11.6.0 software (MedCalc, Mariakerke, Belgium).

## Results

### Patient characteristics

Each of the 16 residents was evaluated at each of the three time points (M1, M3, and M6), for a total of 48 TTEs, in 48 different patients. Table 2 lists the main patient characteristics and TTE findings. Among the 48 patients, no patients had acute respiratory distress syndrome, 45% were receiving catecholamines and 10% neuromuscular blockers at the time of the TTE evaluation.

**Table 2:** Characteristics of the 48 transthoracic echocardiography evaluations

**Patient characteristics**	
Septic shock	26 (54%)
Cardiogenic shock	7 (15%)
Hypovolemic shock	15 (31%)
Norepinephrine	16 (33%)
Epinephrine	4 (8%)
Dobutamine	2 (4%)
Neuromuscular blockers	5 (10%)
**Main TTE findings**	
Decreased left ventricular ejection fraction	15 (31%)
Respiratory variation in IVC diameter	16 (33%)
Non-compressive pericardial effusion	16 (33%)
Right ventricular dilation	8 (17%)

N = 48.

Data are expressed as n (%).

*IVC*: inferior vena cava; *TTE*: transthoracic echocardiography.

### Sensitivity of the scoring system to change

**Figure 1:**
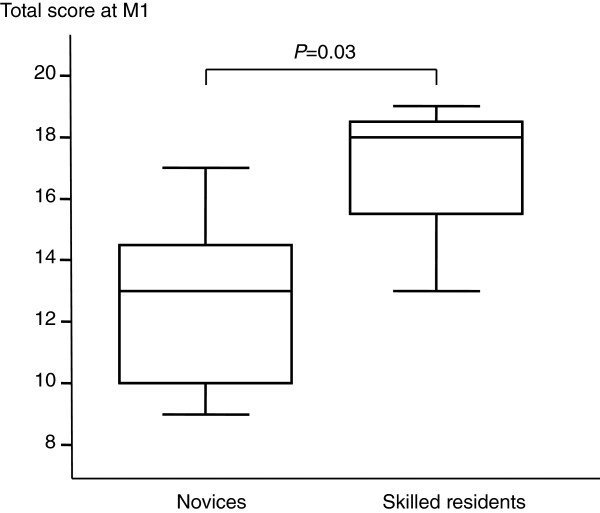
**Total transthoracic echocardiography scores at M1 in the 12 novices and 4 skilled residents.** The box shows the 25th and 75th percentiles, the line in the box the median, and the whiskers the 5th and 95th percentiles.

The median total number of supervised TTEs performed by each novice was 7 (5 to 10) at M1, 27 (24 to 30) at M3 and 67 (57 to 80) at M6 (Table 3). At M1, the median total score was significantly higher in the group of skilled residents than in the group of novices (18 (16 to 19) versus 13 (10 to 15), respectively, *P* = 0.03) (Figure 1).

**Table 3:** Scores for basic critical-care transthoracic echocardiography obtained by the novices at M1, M3, and M6

	**M1**	**M3**	**M6**	***P***-**value**
Number of TTEs performed since start of training	7 (5 to 10)	27 (24 to 30)	67 (57 to 80)	0.0003
Total Score (/20)	13 (10 to 14)	15 (12 to 16)	17 (15 to 18)	< 0.001
Score on the first part of the scoring system (image quality/12)	7 (5 to 9)	8 (7 to 9)	10 (9 to 11)	< 0.001
Score on the first part of the scoring system (semi-quantitative measurements/8)	6 (5 to 7)	7 (5 to 8)	7 (6 to 8)	0.07
Time from starting TTE to suggesting the diagnosis/treatment				
< 15 minutes	0 (0%)	0 (0%)	6 (50%)	0.03
15 to 20 minutes	0 (0%)	4 (33%)	4 (33%)	0.1
< 20 minutes	12 (100%)	8 (67%)	2 (17%)	0.002
Agreement with experts concerning diagnosis/treatment	0.6 ± 0.5	0.5 ± 0.5	0.8 ± 0.4	< 0.0001

N = 12.

Quantitative data are expressed as mean ± standard deviation or median (25% to 75% IQR) range and qualitative data as n (%).

*M*: months; *TTE*: transthoracic echocardiography.

**Figure 2:**
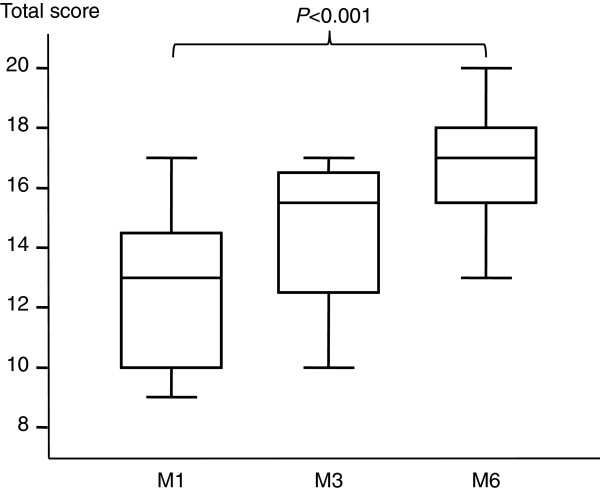
**Total transthoracic echocardiography scores at M1, M3, and M6 in the 12 novices.** The box shows the 25th and 75th percentiles, the line in the box the median, and the whiskers the 5th and 95th percentiles.

In the novices, the total score increased significantly from M1 to M6 (Table 3, Figure 2). The image-quality part of the score increased significantly from M1 to M6, whereas the semi-quantitative measurement part showed a trend toward an increase from M1 to M6 (Table 3). At M6, 83% of novices obtained a total score ≥ 15/20 points.

**Figure 3:**
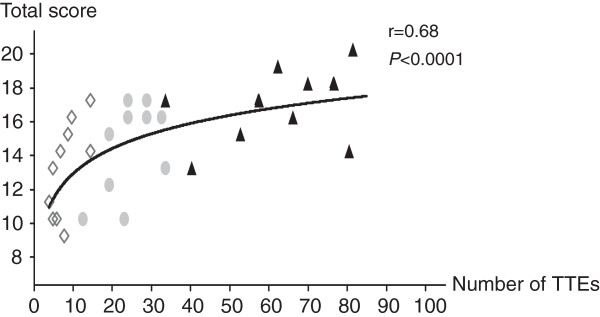
**Correlation between the total transthoracic echocardiography (TTE) score and the number of supervised TTEs performed by the novices at M1, M3, and M6.** N = 33. The line is the correlation line; total scores at M1 are shown as squares, at M3 as circles, and at M6 as triangles.

In the novice group, the percentage of optimal TTE views was significantly higher at M6 than at M1 for the inferior vena cava view (75% versus 17%, respectively; *P* = 0.02) but not for the apical and parasternal views. The number of supervised TTEs correlated significantly with the total TTE score (r = 0.68, *P* < 0.0001) (Figure 3). The proportion of novices who needed less than 15 minutes to perform TTE and who gave the right diagnosis and treatment increased significantly from M1 to M6 (0% versus 50%, respectively, *P* = 0.03) (Table 3).

### Ability of the scoring system to predict a correct diagnosis and treatment

In the group of novices, agreement with the experts regarding the diagnosis/treatment improved significantly from M1 to M6 (Table 3). At M6, 10 (83%) of the 12 novices gave the right diagnosis and treatment.

**Figure 4:**
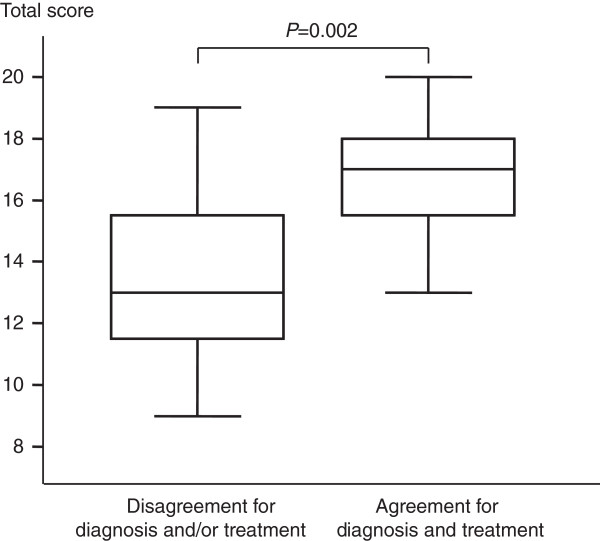
**Total transthoracic echocardiography score according to agreement for both the diagnosis and the treatment between residents (N = 16) and experts.** The box shows the 25th and 75th percentiles, the line in the box the median, and the whiskers the 5th and 95th percentiles.

In the overall population of 16 residents, the total TTE score was significantly higher in the subgroup that gave the right diagnosis and treatment compared to the other subgroup (17 (16 to 18) versus 13 (12 to 16), respectively, *P* = 0.002) (Figure 4).

The ROC curve plotted using all 48 TTE evaluations in the 16 residents showed that a total score of 14/20 was the best cutoff for predicting full agreement regarding the diagnosis and treatment between residents and experts (area under the ROC curve, 0.78; *P* < 0.0001 versus 0.50, sensitivity 81% (95% CI, 64 to 93%) and specificity 69% (95% CI, 41 to 89%)) (Additional file 1). A total score ≥ 19/20 points had 100% specificity (95% CI, 79 to 100%) for full agreement with the experts regarding the diagnosis and treatment.

## Discussion

In this study, we developed and validated a scoring system for assessing basic critical-care TTE skills in ventilated critically ill patients. A cutoff of 19/20 points had 100% specificity for agreement between residents and experts regarding the diagnosis and treatment.

In recent years, echocardiography has gained widespread acceptance among intensivists for the management of acute circulatory failure [29]. Echocardiography allows a rapid evaluation of the hemodynamic status and is recommended as a diagnostic tool in all patients with persistent circulatory failure despite adequate fluid therapy [26]. There is general agreement that echocardiography to assess patients with circulatory failure can be performed reliably by operators who have a lower skill level than that required in cardiology [29]. This basic skill level can be taught easily, as demonstrated in many studies [9-11,20,30-35,42-45]. However, tools are needed to evaluate the skill level achieved by trainees. Experts agree that the skill level does not correlate reliably with the number of supervised echocardiograms [29], as performance could vary with ease of learning, quality of teaching, and specialty of the trainees. Two studies by the same group validated a scoring system for assessing advanced critical-care TEE [37,38].

We developed a score derived from this previously reported TEE scoring system [37,38]. Our score was designed for TTE and assessed basic critical-care skills, which are the ability to measure semi-quantitative parameters but not quantitative parameters [46]. Furthermore, in contrast to the advanced TEE score, our basic TTE score did not include the diagnosis and suggested treatment. We were therefore able to correlate score values to agreement between trainees and experts regarding the diagnosis and treatment, as a means of validating the scoring system. We found that a score cutoff of 19/20 had 100% specificity for full agreement between residents and experts regarding the diagnosis and treatment.

We validated our scoring system in three ways. First, at M1, the total score differed significantly between residents with and without prior echocardiography experience. Second, the total score obtained by novices improved significantly over the six-month teaching period. Third, the total score obtained by the overall population of residents was significantly associated with the ability to make the correct diagnosis and to suggest the appropriate treatment, as defined using the opinion of two experts as the reference standard.

Our findings are consistent with previous evidence that acquiring basic critical-care echocardiography skills requires only a limited amount of training [9-11,30-35]. The theoretical teaching in our study lasted only two hours. By M6, after performing a total of 67 supervised TTEs, 83% of novices obtained scores ≥ 15/20 points and 83% indicated the correct diagnosis and treatment. The time from starting TTE to suggesting a treatment also decreased considerably over the six-month training period. Interestingly, although the novices improved their ability to determine the right diagnosis and treatment between M1 and M6, only the quality of TTE views increased significantly over this period, whereas the improvement in semi-quantitative measurement skills fell short of statistical significance. Therefore, the improved performance regarding diagnosis and treatment is probably ascribable to the accumulation of clinical experience over the six-month rotation rather than to specific improvements in TTE skills. An interesting feature of our study is that supervised TTEs were performed throughout the six-month rotation. This practical approach is easy to use in ICUs that have board-certified echocardiographers on their staff.

The first limitation of our study was that only TTEs in mechanically ventilated patients were considered. This feature improved the standardization of the TTE conditions and increased the time available for teaching and evaluation. However, our score needs to be evaluated in non-intubated patients. Second, the number of residents included in this study was very low. The score cutoffs must therefore be viewed with caution and investigated further to determine whether they are suitable for certification purposes. Third, the theoretical teaching was shorter than the theoretical teaching program currently recommended [29,46] and the hands- on training period was only six months, which is the duration of resident rotations in France. A longer theoretical teaching and a longer period of training might have improved the evaluation of our scoring system. In addition, a significant improvement in semi-quantitative measurements might have been found over a longer period. Fourth, as residents could have access to all patients’ medical folders, they might be influenced before performing their TTE evaluation and we could not exclude the possibility that the number of TTE examinations needed to obtain a satisfactory score might be underestimated. Fifth, residents performed only a single TTE examination during their evaluation, which therefore might not reflect their true TTE proficiencies because of a bias due to the individual patient’s pathology. Nevertheless, all patients with poor echogenicity, as assessed by the experts, were not considered for TTE evaluation. Sixth, the scoring system did not include the exact items described in a statement by two critical-care societies as appropriate for assessing basic skills [46]. This statement was published after our study was designed. Seventh, we did not previously perform a validation study to first assess the scoring system and we cannot state with certainty that two experts was an adequate number to carry out the TTE evaluations. Nevertheless, in our study, two experts evaluated the trainees to avoid the bias induced by only one expert analysis.

## Conclusions

The scoring system that we developed for assessing basic critical-care TTE skills is valid and predicts whether trainees can identify the correct diagnosis and treatment in patients with acute circulatory failure. Further studies are needed to determine whether this score is suitable for critical-care echocardiography certification.

## Abbreviations

CI: confidence interval; IQR: interquartile range; ROC: receiver-operating characteristics; TEE: transesophageal echocardiography; TTE: transthoracic echocardiography.

## Competing interests

The authors declare that they have no competing interests.

## Authors’ contributions

MJ performed analysis and interpretation of the data and drafted the manuscript. XM contributed to analysis and interpretation of the data and to drafting of the manuscript. RC contributed to analysis and interpretation of the data and to drafting of the manuscript. FB performed the collection of data, contributed to analysis and interpretation of the data and helped to draft the manuscript. JR participated in the design of the study, contributed to analysis and interpretation of the data and helped to draft the manuscript. GB conceived the study, performed the collection of data, performed analysis and interpretation of the data and drafted the manuscript. All authors read and approved the final manuscript.

## Supplementary Material

Additional file 1 Receiver-operating characteristics curve generated to identify the score cutoff that predicted full agreement between all residents (n = 16) and experts regarding the diagnosis and treatment. Click here for file
