# Supplementary material for: Prospective assessment of a score for assessing basic critical-care transthoracic echocardiography skills in ventilated critically ill patients
Source: Ann Intensive Care. 2014 Apr 27;4:12. doi: 10.1186/2110-5820-4-12 (PMC4113285; doi:10.1186/2110-5820-4-12)
Supplement: Supplementary file 1 [file 2110-5820-4-12-S1.docx]

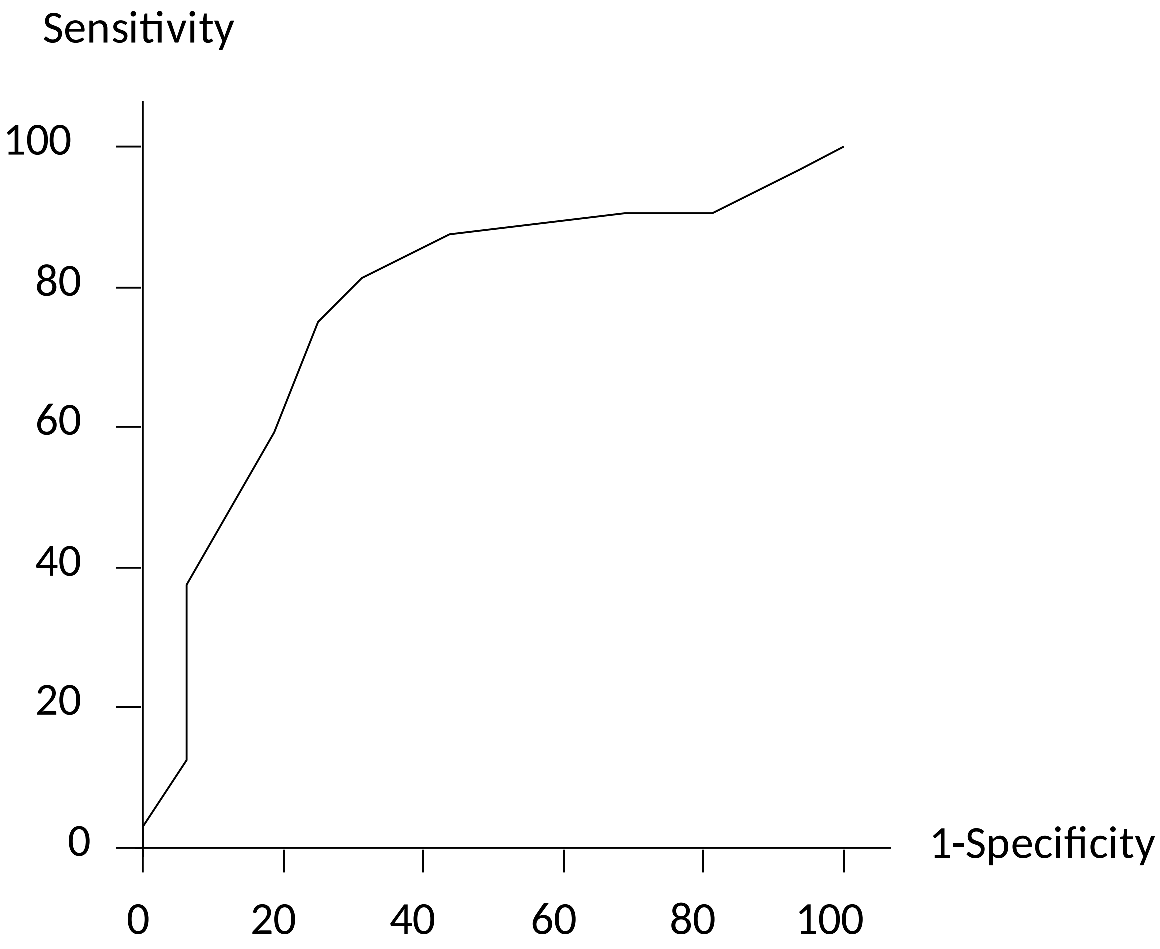

Additional file 1
